# Can multidetector CT replace MRI for evaluating mesorectal fascia in rectal cancer?

**DOI:** 10.37796/2211-8039.1311

**Published:** 2023-03-01

**Authors:** Maryam Farghdani, Mehdi Karami, Amirafraz Fallah Najmabadi

**Affiliations:** a Isfahan University of Medical Sciences, Iran; b Isfahan Uni, Iran

**Keywords:** Fascia, Magnetic resonance imaging, Multidetector computed tomography, Rectal neoplasms

## Abstract

**Background:**

Colorectal cancer is the fourth leading cause of cancer mortality. This study aims to compare the clinical outcomes of multidetector computed tomography (MDCT) with magnetic resonance imaging (MRI) for assessing mesorectal fascia (MRF) in patients with rectal cancer.

**Methods:**

This research was a cross-sectional study of 60 patients with rectal cancer referred to two centers in Isfahan, Al-Zahra, and Seyed-al-Shohada hospitals. Considered parameters included sex, tumoral location, nodal involvement, as well as tumoral description. To assess the invasion of MRF in rectal cancer, researchers used MRI, axial MDCT, and multiplanar reconstruction CT scan (MPRCT). Sensitivity, specificity, and techniques’ positive and negative predictive values were measured. Also, to assess the statistical associations, the Kappa coefficient was used.

**Results:**

There was no significant association between axial MDCT and MRI reports regarding MRF involvement (P>0.05). However, a statistical association was determined between the reports of multiplanar reconstruction CT (MPRCT) and MRI (P< 0.01, kappa=0.44). In addition, the association between MPRCT and MRI reports was statistically significant in patients with wall thickening and negative nodal involvement (Kappa = 0.699, P = 0.001). On the other hand, there was more agreement between MPRCT and MRI reports in patients with tumors in the middle or upper rectum.

**Conclusion:**

The association between MRI and MPRCT reports regarding MRF involvement was statistically significant in patients with wall thickening and negative nodal involvement in the upper and middle rectum. Consequently, it is possible to replace MRI with the MPRCT method for assessing MRF in some patients.

## 1. Introduction

Colorectal cancer is the fourth leading cause of cancer mortality rate and the second most common malignancy worldwide. Approximately one million patients with colorectal cancer are diagnosed annually [1–5], among which nearly 30% of them are detected in the rectal anatomical site [6].

The rectal cancer recurrence rate is higher than colon cancer due to the extensive lymphatic drainage of the pelvis [7]. In comparison to surgery and chemotherapy, which are the traditional treatment methods, neoadjuvant therapy has been recognized as an effective method to reduce the recurrence of the disease and improve the prognosis in recent years. Selection for neoadjuvant therapy is based on the stage of the disease. On the other hand, local staging is critical in managing these patients due to incorporating neoadjuvant chemoradiation into treatment protocol [7]. Although conventional oncological surgery is the ideal treatment for advanced T2 rectal cancer [8], preoperative chemo-radiation improves the prognosis of T3 in patients with mesorectal fascia (MRF) involvement [9,10].

To stage rectal cancer, various methods such as multidetector computed tomography (MDCT) and magnetic resonance imaging (MRI) are common. MRI predicts the depth of tumor invasion by visualizing rectal wall layers and MRF [7,11]. Also, the MRI report has more practical details than the MDCT report for tumoral local staging [12,13]. Nevertheless, modern MDCT methods seem advantageous to MRI for determining distant metastases [14–16].

Multiplanar reconstruction CT (MPRCT) can be potentially beneficial in local staging of rectal cancer and evaluating MRF because MPRCT images can be aligned perpendicular or parallel to the axis of the tumor, similar to MRI imaging [17,18]. Considering that a few studies investigated the diagnostic value of MPRCT and axial CT versus MRI in assessing MRF, this study aimed to understand whether the MDCT report is a replaceable method with the MRI report for evaluating MRF in rectal cancer.

## 2. Materials and methods

### 2.1. Sample selection

This cross-sectional study was conducted on patients with a confirmed diagnosis of rectal cancer referred to the radiology department of two sites, Al-Zahra, and Seyed-al-Shohada hospitals, between October 2017 and May 2020. Included patients were documented as having positive rectal malignancy, determined by the biopsy taken via colonoscopy. Patients were referred to the radiology department before receiving neoadjuvant chemoradiotherapy or surgery.

### 2.2. Procedure

A 128-slice MDCT scanner and MRI (1.5 T) with a phased-array coil were provided to assess the involvement of MRF. The MPRCT images were reconstructed as 2 mm sections. The MRI and MDCT data were evaluated at two-week intervals between two assessments. Also, the radiologist who performed each evaluation was blind to the objectives of the study. Meanwhile, techniques’ sensitivity, specificity, positive predictive value (PPV), and negative predictive value (NPV) were examined.

Based on MDCT results, tumors were classified into different groups based on the anatomical surface (anterior and posterior surface), nodal involvement (negative and positive), location of the tumor (upper, middle, and lower rectum), and lesion description (mass and wall thickening). Anterior and posterior tumors were categorized based on the tumor’s location at 180° anterior and 180° posterior in axial images of MDCT.

Patients were divided into three groups according to their location at MDCT images. The tumors were classified into three groups the lower part, middle part, and upper part, which were 5 cm, 5–10 cm, and >10 cm above the anus. Considering their shape in MDCT, tumors were classified into mass or wall thickening. Moreover, tumors were divided into two subgroups based on the positive or negative report of nodal involvement.

### 2.3. Abdominopelvic MDCT was performed according to the following protocol

KV: 120.

MA: 50–500 (min–max).

Intravenous contrast: 100–150 cc.

Imaging was performed with a 70 s delay after contrast administration.

Oral contrast: neutral contrast (1000 cc water 20–30 min before scan).

### 2.4. Rectal MRI was performed according to the following protocol

T2 HASTE in sagittal, coronal, and axial planes with TR/TE = 2000/80.

T2 FSE in sagittal, coronal, and axial planes with TR/TE = 5000/70.

DWI in the axial plane with TR/TE = 6000/80.

Also, no intravenous contrast was used.

Rectal distention was done with 60 ml ultrasound gel before imaging to improve staging accuracy

### 2.5. Inclusion and exclusion criteria

Inclusion criteria were [1] confirmation of rectal cancer in patients by biopsy [2], no past medical history of performing surgery, chemotherapy, or radiotherapy, related to the current medical condition, rectal cancer, and [3] lack of claustrophobia or fear of closed spaces. Also, patients with incomplete medical records were excluded from the study.

### 2.6. Ethical considerations

Conducting this study is confirmed by the Ethical Committee of Isfahan University of Medical Sciences (ID: IR.MUI.MED.REC.1398.275).

### 2.7. Statistical analysis

SPSS version 22. Kappa coefficient was used for statistical analyses. P < 0.05 was considered statistically significant.

## 3. Results

This research study enrolled 60 patients. The mean participants’ age was 61.2 ± 12.59 years old. Table 1 indicates the frequency of patients with rectal concerning different variables. In addition, the correlation of axial CT and MPRCT with MRI findings is shown in Table 2.

The frequency of patients, with a positive diagnosis of MRF invasion determined by axial CT and MPRCT, was 35 (58.3%) and 45 (75%), respectively. Although there was no association between axial CT and MRI considering MRF invasion (P > 0.05), a weak correlation was determined between MPRCT and MRI about this parameter (P < 0.01) (Table 2).

Table 3 shows the sensitivity, specificity, PPV, and NPV, of MPRCT, which were categorized based on different parameters. Similarly, Table 4 includes the same data but for the axial CT. Table 5 presents the correlation of findings between axial CT and MRI categorized based on a set of parameters. According to these tables, no association was determined between axial CT and MRI findings concerning anatomical surface, description of the lesion, and location (P > 0.05). Likewise, Table 6 includes the results of the statistical analysis of the assumption about the association between the clinical findings of MPRCT and MRI. A moderate association was determined between the findings of MPRCT and MRI regarding two parameters, negative nodal involvement as well as the location of the tumor in the upper and middle parts, in both male and female patients (P < 0.05).

Moreover, a significant agreement was determined between the MPRCT and MRI in tumors described as wall thickening (P < 0.05). Similarly, no association was identified between the findings of MPRCT and MRI in tumors located in the lower rectum and tumors with nodal involvement.

## 4. Discussion

MRI is the gold standard method in the prediction of tumor invasion to MRF before chemo-radiation [19–21]. The other method, CT scan, is part of the routine practice in staging patients with rectal cancer. However, the main limitation of staging with the CT scan technique is the inherent poor tissue contrast, in comparison to MRI [17].

For assessing MRF invasion, thin CT scan slices, with comparable accuracy to MRI, is replaceable with MRI, at least for some patients. To the best of our knowledge, a limited number of studies have been conducted on the same objective, assessing the diagnostic value of MDCT, in comparison to MRI, in the diagnosis of MRF invasion in patients with rectal cancer [14]. In addition, considering MRF invasion, a moderate association between the reports of MPRCT and MRI, (k = 0.44, p < b 0.01) was identified. According to the statistical analysis of this study, applying MPRCT for assessing the MRF invasion had more benefits than axial CT did.

Furthermore, by classifying patients into multiple subgroups, we tried to identify whether a significant statistical association is identifiable between MDCT and MRI in any of our subgroups. The findings indicated a moderate association between MPRCT and MRI methods in patients without nodal involvement. However, no association was identified between these methods in patients with nodal involvement.

Among all subgroups, a significant association was identified between the findings of MPRCT and MRI in the group of patients described as wall thickening of the rectum (k = 0.699). In addition, there was a moderate association between the findings of MPRCT and MRI in patients with tumors located at the middle and upper rectum. Vliegen et al. assessed the accuracy of MDCT and MRI in patients with rectal cancer. They reported that the performance of CT scan in the middle and upper rectum was significantly better than in the lower parts [19]. Similarly, according to the findings of this study, the accuracy of MDCT was poor in predicting MRF involvement in tumors that are identified the in lower and anterior parts.

Based on our findings in this study, MPRCT had a stronger association with MRI in patients with tumors described as wall thickening and negative nodal invasion. In detail, the MPRCT reports, which included information about tumors described as wall thickening, located in the upper and middle rectum, and negative nodal invasion, were more consistent with MRI reports for a similar clinical condition. This result may be due to the greater distance of these tumors from the MRF and the possibility of easier differentiation of invasion or non-invasion of this fascia. According to the results, replacing MRI with MPRCT in some patients can be considered in the future. It also benefits patients by a significant decrease in patients’ diagnostic costs.

## 5. Conclusion

According to the results of this study, although axial MDCT is not replaceable with MRI for assessing MRF invasion in patients with rectal cancer, MPRCT showed applicability for this purpose. Moreover, an association was identified between the reports of MRI and MPRCT for assessing MRF invasion in patients with wall thickening of the upper and middle rectum and negative nodal involvement. Hence, MPRCT, which is a common method in assessing distant metastasis, can replace MRI to assess MRF invasion in patients with wall thickening as tumoral description located in the upper and middle rectum and with negative nodal involvement.

## Figures and Tables

**Table 1:** The frequency of patients with rectal carcinoma regarding variables.

Variables	Frequency (Percent)
**Sex**	35 (58.3)
Male	25 (41.7)
Female	60 (100)
Total	
**MRI**	38 (63.3)
Not involved MRF	22 (36.7)
Involved MRF	60 (100)
Total	
**Axial CT**	37 (61.7)
Not involved MRF	23 (38.3)
Involved MRF	60 (100)
Total	
**MPRCT**	41 (68.3)
Not involved MRF	19 (31.7)
Involved MRF	60 (100)
Total	
**Nodal involvement**	28 (46.7)
Negative	23 (38.3)
Positive	60 (100
Total	
**Anatomical surface**	28 (46.7)
Anterior	20 (33.3)
Posterior	48 (80)
Total	12 (20)
Missing value	
**Location**	12 (20)
High	21(35)
Middle	15 (25)
Low	48 (80)
Total	12 (20)
Missing value	
**Lesion**	22 (36.7)
Wall thickening mass	27(45)
Total	49 (81.7)
Missing value	11 (18.3)

**Table 2:** Agreement between MRI with axial. CT and MPRCT.

CT Methods	MRI		Kappa	P-value

	MRF not involved	MRF involved		
Axial. CT	25 (67.5)	12 (32.4)	0.11	0.38
Not involved MRF	13 (56.5)	10 (43.4)		
Involved MRF	38 (63.3)	22 (36.7)		
Total				
MPRCT	32 (78.04)	9 (22)	0.44	0.001
Not involved MRF	6 (31.6)	13 (68.4)		
Involved MRF	38 (63.3)	22 (36.7)		
Total				

**Table 3:** The sensitivity, specificity, PPV and NPV of MPRCT method in terms of variables.

Variables	PPV	NPV	SEN	SP
**Sex**	71.4	72.2	50	86.6
Female	66.6	82.6	66.6	82.6
Male				
**Nodal involvement**	71.4	85.7	62.5	90
Negative	66.6	71.4	60	76.9
Positive				
**Anatomical surface**	60	77.7	60	77.7
Anterior	80	80	57.14	92.3
Posterior				
**Description of Lesion**	100	89.4	60	100
Wall thickening	58.3	66.6	58.3	66.6
Mass				
**Location**	100	80	50	100
High	62.5	92.3	83.3	80
Middle	60	60	42.8	75
Low				

PPV: positive predictive value; NPV: negative predictive value; MPRCT: Multiplanar reconstruction. CT.

**Table 4:** The sensitivity, specificity, PPV, and NPV of axial CT method in terms of variables.

Variables	PPV	NPV	SEN	SP
**Sex**	37.5	58.8	30.0	66.6
Female	46.6	75	58.3	65.2
Male				
**Nodal involvement**	40	77.7	50	70
Negative	45.4	58.3	50	53.4
Positive				
**Anatomical surface**	41.6	68.7	50	61.1
Anterior	50	71.4	42.8	76.9
Posterior				
**Lesion**	33.3	81.2	76.4	…
Wall thickening	46.1	57.1	50	53.3
Mass				
**Location**	50	75	50	75
High	45.4	90	83.3	60
Middle	33.3	50	14.2	75
Low				

PPV: positive predictive value; NPV: negative predictive value.

**Table 5:** Agreement between axial CT and MRI findings in terms of variables.

Axial CT in terms of variables		MRI		Kappa	p-value

		Not involved MRF	involved MRF		
Sex	**Female**			−0.34	0.86
	Not involved MRF	10 (58.8)	7 (41.2)		
	involved MRF	5 (62.5)	3 (37.5)		
	**Male**			0.22	0.18
	Not involved MRF	15 (75)	5 (25)		
	involved MRF	8 (53.3)	7 (46.6)		
Nodal involvement	**Negative**			0.186	0.318
	Not involved MRF	14 (77.7)	4 (22.22		
	involved MRF	6 (60)	4 (40)		
	**Positive**			0.038	0.855
	Not involved MRF	7 (58.3)	5 (41.7)		
	involved MRF	6 (54.5)	5 (45.4)		
Anatomical surface	**Anterior**			0.10	0.569
	Not involved MRF	11 (68.7)	5 (31.3)		
	involved MRF	7 (58.3)	5 (41.6)		
	**Posterior**			0.20	0.357
	Not involved MRF	10 (71.4)	4 (28.6)		
	involved MRF	3 (50)	3 (50)		
Description of Lesion	**Wall thickening**			0.15	0.46
	Not involved MRF	13 (81.2)	3 (18.8)		
	involved MRF	4 (66.7)	2 (33.3)		
	**Mass**			0.033	0.86
	Not involved MRF	8 (57.1)	6 (42.9)		
	involved MRF	7 (53.8)	6 (46.1)		
Location	**High**			0.25	0.386
	Not involved MRF	6 (75)	2 (25)		
	involved MRF	2 (25)	2 (25)		
	**Middle**			0.34	0.072
	Not involved MRF	9 (90)	1 (10)		
	involved MRF	6 (54.5)	5 (45.4)		
	**Low**			−0.1	0.60
	Not involved MRF	6 (50)	6 (50)		
	involved MRF	2 (66.7)	1(33.3)		

**Table 6:** Agreement between the findings of MPRCT and MRI in terms of variables.

MPRCT in terms of variables		MRI		Kappa	P-value
	
Variables		No	Yes		
Sex	**Female**				
	Not involved MRF	13 (72.2)	5 (27.8)	0.38	0.045
	involved MRF	2 (28.6)	5 (71.4)		
	**Male**				
	Not involved MRF	19 (82.6)	4 (17.4)	0.49	0.004
	involved MRF	4 (33.3)	8 (66.6)		
Nodal involvement	**Negative**				
	Not involved MRF	18 (85.7)	3 (14.3)	0.54	0.004
	involved MRF	2 (28.6)	5 (71.4)		
	**Positive**				
	Not involved MRF	10 (71.4)	4 (28.6)	0.37	0.072
	involved MRF	3 (33.3)	6 (66.6)		
Anatomical surface	**Anterior**				
	Not involved MRF	14 (77.7)	4 (22.2)	0.37	0.046
	involved MRF	4 (40)	6 (60)		
	**Posterior**				
	Not involved MRF	12 (80)	3 (20)	0.52	0.015
	involved MRF	1 (20)	4 (80)		
Description of Lesion	**Wall thickening**				
	Not involved MRF	17 (89.4)	2 (10.5)	0.699	0.001
	involved MRF	0 (0)	3 (100)		
	**Mass**				
	Not involved MRF	10 (66.6)	5 (33.3)	0.25	0.194
	involved MRF	5 (41.7)	7 (58.3)		
Location	**High**				
	Not involved MRF	8 (80)	2 (20)	0.57	0.028
	involved MRF	0 (0)	2 (100)		
	**Middle**				
	Not involved MRF	12 (92.3)	1 (7.7)	0.57	0.007
	involved MRF	3 (37.5)	5 (62.5)		
	**Low**				
	Not involved MRF	6 (60)	4 (40)	0.24	0.464
	involved MRF	2 (40)	3 (60)		

## References

[1] Raman S Chen Y Fishman E Evolution of imaging in rectal cancer: multimodality imaging with MDCT, MRI, and PET J Gastrointest Oncol 2015 6 2 172 84 25830037 10.3978/j.issn.2078-6891.2014.108 PMC4311095

[2] Samee A Selvasekar CR Current trends in staging rectal cancer World J Gastroenterol 2011 17 828 34 21412492 10.3748/wjg.v17.i7.828 PMC3051133

[3] Fowler K Kaur H Cash B Feig B Gage K Garcia E ACR Appropriateness Criteria pretreatment staging of colorectal cancer J Am Coll Radiol 2012 9 775 981 23122343 10.1016/j.jacr.2012.07.025

[4] Sabori S Esmaeli H Shahid Saleh S Determining related factors to survival of colorectal cancer patients using cox regression Shahid Sadoughi Univ Med Sci 2018 1 9

[5] limelius BG Tiret E Cervantes A Feig B Gage K Garcia E Rectal cancer: ESMO Clinical Practice Guidelines for diagnosis, treatment, and follow-up Ann Oncol 2013 24 vi81 v88 24078665 10.1093/annonc/mdt240

[6] Teama A Abdelsamie Alarabawy R Abdelh ady Mohamed H Hany Eissa H Role of magnetic resonance imaging in assessment o,f rectal neoplasms Egypt J Radiol Nucl Med 2015 46 4 833 46

[7] Rao Sh-X Zeng M Xu J-M Qin X-Y Chen C Assessment of T staging and mesorectal fascia status using high-resolution MRI in rectal cancer with rectal distention World J Gastroenterol 2007 13 30 4141 6 17696238 10.3748/wjg.v13.i30.4141 PMC4205321

[8] Borschitz T Wachtlin D Möhler M Neoadjuvant chemoradiation and local excision for T2-3 rectal cancer Ann Surg Oncol 2008 15 3 712 20 18163173 10.1245/s10434-007-9732-x

[9] Kapiteijn E Marijnen CA Nagtegaal ID Putter H Steup WH Preoperative radiotherapy combined with total mesorectal excision for resectable rectal cancer N Engl J Med 2001 345 638 46 11547717 10.1056/NEJMoa010580

[10] Colorectal Cancer Collaborative Group Adjuvant radiotherapy for rectal cancer: a systematic overview of 8,507 patients from 22 randomized trials Lancet 2001 358 1291 304 11684209 10.1016/S0140-6736(01)06409-1

[11] Poon FW McDonald A Anderson JH Duthie F Rodger C McCurrach G Accuracy of thin section magnetic resonancrandomizedased-array pelvic coil in predicting the T-staging of rectal cancer Eur J Radiol 2005 53 256 62 15664289 10.1016/j.ejrad.2004.03.011

[12] Mathur P Smith JJ Ramsey C Owen M Thorpe A Karim S Comparison of CT and MRI in the pre-operative staging of rectal adenocarcinoma and prediction of circumferential resection margin involvement by MRI Colorectal Dis 2003 5 5 396 401 12925069 10.1046/j.1463-1318.2003.00537.x

[13] Singla S Kaushal D Singh Sagoo H Calton N Comparative analysis of colorectal carcinoma staging using operative, histopathology and computed tomography findings Int J Appl Basic Med Res 2017 7 1 10 4 28251101 10.4103/2229-516X.198501 PMC5327599

[14] Ippolito D Girolama Drago S Talei Franzesi C Fior D Siron S Rectal cancer staging: multidetector-row computed tomography diagnostic accuracy in assessment of mesorectal fascia invasion World J Gastroenterol 2016 22 20 4891 900 27239115 10.3748/wjg.v22.i20.4891 PMC4873881

[15] Wolberink SV Beets-Tan RG De Haas-Kock DF Van de Jagt EJ Span MM Wiggers T Multislice CT as a primary screening tool for the prediction of an involved mesorectal fascia and distant metastases in primary rectal cancer: a multicenter study Dis Colon Rectum 2009 52 928 34 19502858 10.1007/DCR.0b013e318194f923

[16] Ahmetoğlu A Cansu A Baki D Kul S Cobanoğlu U Alhan E MDCT with multiplanar reconstruction in the preoperative local staging of the rectal tumor Abdom Imag 2011 36 31 7 10.1007/s00261-009-9591-y 19949791

[17] Sinhaa R Vermaa R Rajesha A Richards C Diagnostic value of multidetector row CT in rectal cancer staging: comparison of multiplanar and axial images with histopathology Clin Radiol 2006 61 924e931 34 17018304 10.1016/j.crad.2006.03.019

[18] Kwok H Bissett IP Hill GL Preoperative staging of rectal cancer Int J Colorectal Dis 2000 15 9e20 5 10766086 10.1007/s003840050002

[19] Vliegen R The accuracy of Multi-detector row CT for the assessment of tumor invasion of the mesorectal fascia in primary rectal cancer Abdom Imag 2008 604 610 10.1007/s00261-007-9341-y PMC2491404 18175167

[20] Son I Oncologic relevance of magnetic resonance imaging–detected threatened mesorectal fascia for patients with mid or low rectal cancer: a longitudinal analysis before and after long-course, concurrent chemoradiotherapy Surgery 2017 1 9 10.1016/j.surg.2017.01.011 28237642

[21] Beets-Tan RG Beets GL Rectal cancer: review with emphasis on MR imaging Radiology 2004 232 335 46 15286305 10.1148/radiol.2322021326

